# The Impact of Internet Hospital Follow-Up on the Quality of Life of Patients With Epilepsy: Randomized Controlled Trial

**DOI:** 10.2196/70665

**Published:** 2025-05-26

**Authors:** Ting Ting Liu, Min Zhang, Hong Ying Li, Yu Wen Zhang, Lu Liang, Xin Yue Huang, Xia Gan, Lan Mou, Chen Shi Liu, Ming Ming Zhang, Jie Liu

**Affiliations:** 1 Department of Medical Record and Statistics Sichuan Academy of Medical Sciences & Sichuan Provincial People’s Hospital University of Electronic Science and Technology of China Chengdu China; 2 Department of Outpatient Sichuan Academy of Medical Sciences & Sichuan Provincial People’s Hospital University of Electronic Science and Technology of China Chengdu China; 3 Department of Neurology Sichuan Academy of Medical Sciences & Sichuan Provincial People’s Hospital University of Electronic Science and Technology of China Chengdu China; 4 Department of Center of Health Physical Examination and Health Management Sichuan Academy of Medical Sciences & Sichuan Provincial People’s Hospital University of Electronic Science and Technology of China Chengdu China

**Keywords:** epilepsy, internet hospital, quality of life in patients with epilepsy, anxiety, depression, chronic disease management of epilepsy

## Abstract

**Background:**

As the second most common neurological disorder, epilepsy requires long-term management to ensure better seizure control and improved patient outcomes. Health education and sustained care significantly help people with epilepsy manage their condition effectively. Internet hospitals (IHs) have emerged as a promising approach to enhancing health care accessibility. These digital platforms can significantly improve the quality of life for patients with epilepsy. However, despite their growing adoption, research on the application of IHs in the follow-up management of epilepsy remains limited, highlighting the need for further investigation.

**Objective:**

This study has 2 primary aims. The first aim was to assess and compare the differences in quality of life, anxiety, and depression between IH follow-up and traditional outpatient follow-up for patients with epilepsy. The second aim was to explore chronic disease management models that are tailored to meet the needs of patients with epilepsy, improving their overall care.

**Methods:**

Eligible patients diagnosed with epilepsy were recruited at Sichuan Provincial People's Hospital and randomly assigned to the intervention or control group. Data collected included demographic information, clinical characteristics, and scores from the Quality of Life in Epilepsy-31 (QOLIE-31), Generalized Anxiety Disorder-7 Scale (GAD-7), and Neurological Disorders Depression Inventory for Epilepsy (NDDI-E). The control group received traditional outpatient follow-up, while the intervention group was managed via the IH. Both groups received epilepsy health education. After 6 months, changes in quality of life, anxiety, and depression were assessed using the same scales. Data analysis followed the intention-to-treat principle, and a linear mixed model was used to examine the intervention effect on primary and secondary outcomes. The effect sizes of group differences were calculated using Hedges *g* (0.2-0.4: small, 0.5-0.7: medium, and ≥0.8: large).

**Results:**

A total of 214 patients with epilepsy participated in the study (intervention group: N=107; control group: N=107). At the end of the study, 94.4% (101/107) in the intervention group and 93.5% (100/107) in the control group had completed the follow-up visits. After the intervention, the intention-to-treat analysis revealed evidence for improved quality of life (QOLIE-31 total score; *F*_216.53_=13.10, *P*<.001) with small between-group effects (Hedges *g*=0.49, 95% CI 0.22-0.76) in favor of the intervention group. We also found evidence of reduced depression, as well as improved seizure worry, overall quality of life, emotional well-being, energy or fatigue, medication side effects, with effects ranging from small to medium (Hedges *g*=0.42-0.79).

**Conclusions:**

Long-term follow-up through IHs can effectively improve the quality of life and reduce anxiety and depression in patients with epilepsy. This model provides effective support, making it an important tool for managing epilepsy. Therefore, IH management is recommended as a feasible approach for epilepsy follow-up in clinical practice.

**Trial Registration:**

Chinese Clinical Trial Registry ChiCTR2500101061; https://www.chictr.org.cn/showprojEN.html?proj=260855

## Introduction

Epilepsy, classified by the World Health Organization as one of the 5 major neuropsychiatric disorders, affects approximately 10 million people in China, with a prevalence of 0.9‰ to 8.5‰ [1]. The primary treatment goal is seizure control and frequency reduction, with antiseizure medications as the first-line therapy. However, about one-third of patients develop drug-resistant epilepsy (DRE), defined as the failure to achieve sustained seizure freedom despite trials of at least 2 well-tolerated, appropriately chosen, and correctly administered antiseizure drug regimens, whether as monotherapies or in combination [2]. The chronic nature of this condition severely impacts patients' daily lives, as they not only struggle with cognitive impairment caused by seizures but also face challenges such as drug resistance, adverse drug reactions, and comorbid anxiety and depression. Previous studies have shown that the quality of life for patients with epilepsy is significantly lower than that of individuals with other chronic diseases and the general population [3]. The quality of life for patients with epilepsy is influenced by various factors, including seizure frequency, comorbid anxiety and depression, and side effects of medication [4]. Anxiety and depression in patients with epilepsy are significantly negatively correlated with their quality of life [5,6]. Therefore, improving the overall quality of life is crucial for patients with epilepsy.

Internet health care represents an emerging model of medical services that integrates advanced technologies such as cloud computing, the Internet of Things, big data, and mobile communications, with the internet as its core platform [7]. Unlike traditional in-person consultations, internet health care offers enhanced convenience by transcending time and geographical constraints, providing services such as telemedicine and internet hospitals (IHs) [8]. Telemedicine is defined as “the use of medical information exchanged from one site to another via electronic communications to improve a patient’s clinical health status” [9]. It encompasses various medical activities, including remote diagnosis, remote consultations and nursing, remote education, and the provision of remote medical information services [10]. IH refers to hospital-based service platforms that integrate consultation, prescription, payment, and drug delivery, mostly featured in digital follow-up and routine consultations [11]. IHs represent an innovative business model emerging from China’s “Internet +” health care initiative, acting as an extension of both telemedicine and conventional brick-and-mortar hospitals. By integrating the medical resources of physical hospitals with internet technologies, IHs offer a seamless combination of web-based and in-person services, as well as front-end and back-end medical support. Patients can communicate with doctors remotely via the platform, access their medical records, prescriptions, and follow-up plans. The current service quality of Chinese IHs as perceived by patients did not yet meet their expectations, especially in terms of service responsiveness [11]. In general, the concept of IHs can cover telemedicine [12]. IHs not only perform the functions of telemedicine but also carry out certain roles of traditional physical hospitals. IHs can provide patients with a full range of web-based medical and nonmedical services related to outpatient care and hospitalization, whereas telemedicine mainly offers medical-related services such as remote diagnosis, remote consultations, and remote education [13]. The COVID-19 pandemic accelerated the growth of IHs in China, with these platforms addressing challenges such as limited access to in-person care, time constraints, and geographic barriers to health care.

Long-term management of epilepsy necessitates sustained care and health education. Traditional outpatient follow-up models, while effective, are often constrained by geographic barriers, time costs, and limited accessibility to specialized care. Telemedicine, telephone consultations, mobile health applications, and platforms like WeChat—have emerged as supplementary approaches to address these challenges. For example, studies have demonstrated improved treatment adherence in pediatric patients with epilepsy through WeChat-based follow-up programs [14]. However, the aforementioned models are not connected to physical hospitals. Previous studies have shown that IHs, in terms of medical experience, are comparable to offline physical hospitals and can effectively establish a physician-patient relationship similar to that of offline physical hospitals [12].

Nevertheless, research on the impact of IH follow-up on quality of life, anxiety, and depression among patients with epilepsy remains limited; the effectiveness of IHs for epilepsy patients has not been confirmed. This study aims to compare IH follow-up management with traditional outpatient follow-up in terms of quality of life, anxiety, and depression among patients with epilepsy, with the goal of exploring innovative follow-up management models tailored to their needs. We hypothesize that IH follow-up can improve quality of life and alleviate anxiety and depression in patients with epilepsy.

## Methods

### Study Setting

This single-center, parallel-group, randomized controlled trial was conducted in accordance with the principles of the Declaration of Helsinki.

### Ethical Considerations

This study was approved by the Institutional Ethics Board of Sichuan Provincial People's Hospital (number 2023-587). All individuals meeting the inclusion criteria provided informed consent after being briefed on the study’s objectives and procedures and then completed the questionnaire unaided. To ensure confidentiality, each participant’s name was replaced by a computer‑generated code, and no material or financial incentives were provided. This study was registered with the Chinese Clinical Trials Registry (ChiCTR2500101061).

### Participants

Study participants were conveniently recruited from the Sichuan Provincial People's Hospital outpatient practice. Participants were enrolled from November 2023 to April 2024.

The inclusion criteria were as follows: participants were aged 18 years or older; according to the 2014 definition by the International League Against Epilepsy, patients who were previously diagnosed with epilepsy and received medication treatment, with seizure types classified according to the 2017 International League Against Epilepsy classification into focal seizures, generalized seizures, and others [15]; able to understand the content of the questionnaires and complete them independently; patients and their families were informed of the study details, consented to participate, understood the purpose and significance of the research, and agreed to the use of relevant data for scientific purposes.

The exclusion criteria were as follows: presence of abnormal mental behavior or a history of diagnosed mental illness; recent severe trauma, such as significant family upheaval or undergoing surgical procedures; presence of other conditions that may affect quality of life, such as malignant tumors or cerebrovascular diseases; and DRE [16]: lack of success in achieving sustained seizure freedom despite trials of 2 tolerated, appropriately selected, and correctly administered antiepileptic drug regimens (whether as monotherapies or in combination).

### Allocation and Blinding

Participants will be randomly assigned to groups using a simple randomization method based on computer-generated random numbers, ensuring an equal distribution across groups. The randomization process will be overseen by a designated trial team member. After the participant sequence table is prepared, individuals will be enrolled using a consecutive sampling method. Once randomization is completed, the principal investigator will disclose the allocation and inform the respective investigators in each study arm, ensuring that blinding is maintained prior to allocation. Due to the nature of the intervention, both investigators and participants will be aware of their group assignments. However, data analysis will be conducted independently by 2 statisticians who remain blinded to group allocation to minimize bias.

#### Intervention

The follow-up team consisted of 2 nurses with more than 5 years of experience in epilepsy nursing and 2 epileptologists. The health education content was primarily delivered through pamphlets with animations, explaining various aspects of epilepsy. The educational program covered several key topics, including disease knowledge, epilepsy treatment guidelines, healthy living, balanced diet, and psychological guidance. Epilepsy nurses provided one-on-one structured teaching to ensure all aspects of epilepsy care were covered. Each session lasted at least 30 minutes, and each patient randomly assigned to the epilepsy health education group received a total of 6 sessions. It is important to note that this program was intentionally designed to differ from well-established, more intensive programs like the MOSES (Modular Service Package Epilepsy) program [17]. While the MOSES program is typically more intensive, our program aims to create an intervention that can be implemented both in-person and web-based, ensuring it is scalable and flexible. The epilepsy nurses provided one-on-one teaching in a structured format, addressing all these aspects of epilepsy. Each session lasted at least 30 minutes, with a total of 6 sessions for each patient randomly assigned to the epilepsy health education group.

The control group received regular follow-up management in the outpatient department, while the intervention group received follow-up management through the IH. Patients with epilepsy in the outpatient follow-up program received face-to-face follow-up and health education at the outpatient department of Sichuan Provincial People's Hospital. In contrast, patients with epilepsy in the IH follow-up program could interact with health care providers through web-based consultations or video consultations using methods such as videos, images, and text via the Sichuan Provincial People's Hospital Internet Hospital platform. A fixed day each month will be scheduled for follow-up visits and epilepsy health education for patients with epilepsy. Patients with epilepsy will be assessed for quality of life, anxiety, and depression in both groups at the beginning of the study, and after 6 months, patients will be reassessed to compare changes in quality of life, anxiety, and depression between the 2 groups.

#### Primary Outcome

We defined the quality of life as the primary outcome, measured by the American Quality of Life in Epilepsy-31 (QOLIE-31) scale, assessed at baseline and 6 months after randomization. The American QOLIE-31, developed by Cramer et al [18] in 1998, was used to assess the quality of life. The QOLIE-31 includes 7 subdomains: seizure worry, overall quality of life, emotional well-being, energy or fatigue, cognition, medication side effects, and social function. The scale includes a total of 31 items, which collectively evaluate the overall health status.

#### Secondary Outcomes

The secondary outcomes were Generalized Anxiety Disorder-7 Scale (GAD-7) and Neurological Disorders Depression Inventory for Epilepsy (NDDI-E), measured at baseline, 6 months after randomization.

The GAD-7 scale was used to assess anxiety levels [19]. It consists of 7 items, each scored on a 4-point scale ranging from 0 to 3. The total score of the GAD-7 is 21, with higher scores indicating more severe anxiety symptoms.

NDDI-E is a self-assessment tool consisting of 6 questions. By evaluating depressive symptoms over the past 2 weeks, it can quickly identify whether patients with epilepsy have depressive disorders. It is widely used to assess depression in patients with epilepsy. Each question is scored on a range of 1 to 4 points, with a total score range of 6 to 24 points [20].

#### Data on Demographic and Clinical Characteristics

Demographic data included age, gender, education level, family economic status, and household registration type. Clinical data covered seizure type, seizure frequency in the past year, number of antiepileptic drugs, age at first seizure, and duration of epilepsy.

### Sample Size

The sample size estimation was based on the primary outcome—QOLIE-31 scale. Based on literature review, a minimal detectable difference of 5 points was selected, and the estimated SD of the QOLIE-31 scale score is 10 [21]. The significance level (α) was defined as 0.05, β as 0.1, and the desired statistical power was 90%. Using the formula for sample size calculation, the estimated minimum sample size per group is 86 participants. After considering a 10% dropout rate, it was decided to include 96 participants in both the control and intervention groups.

### Data Processing and Analysis

All data analyses were conducted using an intention-to-treat approach, including all participants, with SPSS (version 25.0; IBM Corp) Baseline data were presented as means and SDs for continuous variables and as counts and percentages for categorical variables. Differences between randomization groups were assessed using independent sample 2-tailed *t* tests for continuous variables and chi-square tests for categorical variables. Missing values during follow-up were handled using multiple imputations by predictive mean matching. For primary and secondary outcomes, a linear mixed model analysis was applied to compare the intervention effects between groups. The models incorporated group, time, and interactions between group and time as fixed covariates and the participants as random intercepts. Multiple contrasts were adjusted using a Bonferroni post hoc test. Effect sizes (Hedges *g*) for within-group and between-group differences were calculated using the pooled standard deviation of complete cases only and were classified as small (Hedges *g*=0.2), medium (Hedges *g*=0.5), and large (Hedges *g*=0.8).

## Results

### Participants’ Characteristics

**Figure 1:**
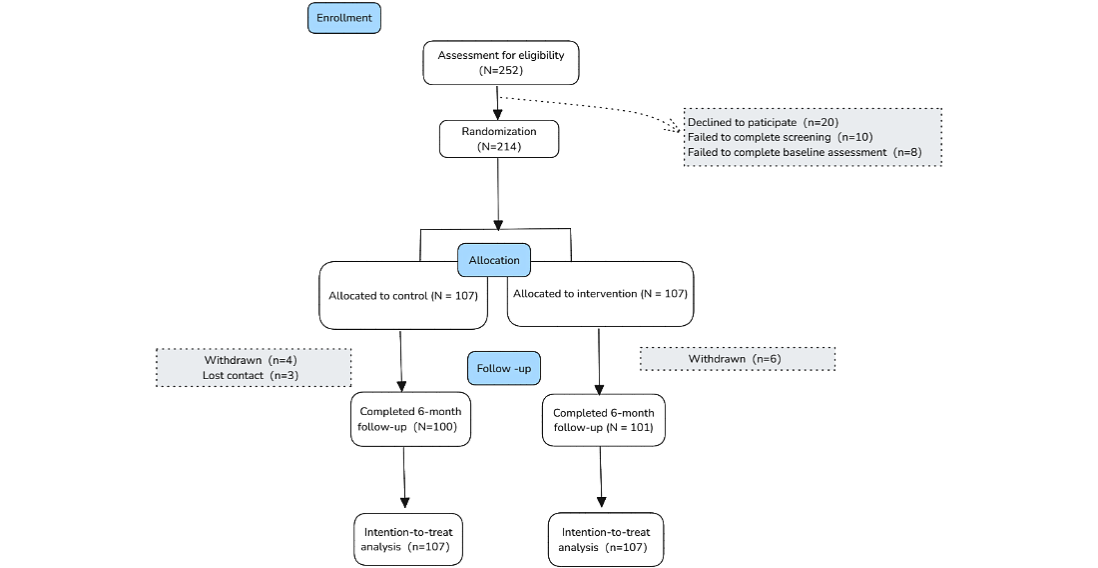
CONSORT flowchart.

The follow-up assessment ended in November 2024. The CONSORT (Consolidated Standards of Reporting Trials) diagram of the participant flow is shown in Figure 1 and the checklist is provided in Multimedia Appendix 1. A total of 252 participants were screened for eligibility, from which 38 (15.1%) participants were excluded: 20 (7.9%) declined to participate, 10 (3.9%) failed to complete screening, and 8 (3.1%) did not complete baseline assessment. In total, 214 (84.9%) individuals completed the baseline survey and were randomized into the intervention (N=107) and control (N=107) groups. At the end of the study, 94.4% (101/107) in the intervention group and 93.5% (100/107) in the control group had completed the follow-up visits. The demographic, obstetrical characteristics, and outcomes of the participants at baseline were similar between the 2 groups (Table 1).

**Table 1:** Baseline characteristics of participants in the intervention and control groups.

			Intervention group (N=107)		Control group (N=107)		*P* value		
Age (years), mean (SD)			34.02.13 (12.37)		33.81 (12.12)		.43		
Age at first onset (years), mean (SD)			24.90 (13.56)		23.36 (14.566)		.33		
Duration of illness (years), mean (SD)			9.12 (9.79)		10.45 (10.16)		.90		
**Sex, n (%)**								.10	
	Male	45 (43)		57 (51.5)					
	Female	62 (57)		50 (48.5)					
**Household registration type, n (%)**								.33	
	Rural	57 (56)		64 (65.3)					
	Nonrural	50 (44)		43 (34.7)					
**Educational level, n (%)**								.48	
	＜12 years	38 (34)		43 (40.6)					
	≥12 years	69 (34)		64 (59.4)					
**Income (month/person), n (%)**								.34	
	＜3000 yuan (<US $416.12)	56 (54)		49 (43.6)					
	≥3000 yuan (≥US $416.12)	51 (46)		58 (56.4)					
**Number of drugs, n (%)**								.57	
	1	41 (37)		37 (32.8)					
	≥2	66 (63)		70 (67.3)					
**Type of seizure, n (%)**								.31	
	Focal onset	13 (11)		18 (17.8)					
	Generalized onset	82 (78)		72 (66.3)					
	Unknown	12 (11)		17 (15.8)					
**Seizure frequency, n (%)**								.12	
	≥1/year	60 (63.6)		71 (68.2)					
	＜1/year	47 (36.4)		36 (31.8)					
QOLIE-31^a^ total score, mean (SD)			65.22 (7.21)		65.23 (8.09)		.99		
Seizure worry, mean (SD)			58.33 (13.72)		59.01 (13.82)		.72		
Overall quality of life, mean (SD)			72.21 (11.35)		75.33 (30.29)		.47		
Emotional well-being, mean (SD)			65.42 (8.69)		64.21 (9.85)		.34		
Energy or fatigue, mean (SD)			60.13 (10.37)		60.40 (10.51)		.85		
Cognition, mean (SD)			64.94 (11.96)		64.29 (12.21)		.69		
Medication side effects, mean (SD)			53.67 (14.63)		50.07 (13.90)		.07		
Social Function, mean (SD)			66.18 (9.02)		65.49 (10.911)		.62		
GAD-7^b^ score, mean (SD)			7.43 (5.95)		7.17 (5.28)		.73		
NDDI-E^c^ score, mean (SD)			13.04 (5.63)		12.18 (5.10)		.24		

^a^QOLIE-31: Quality of Life in Epilepsy-31.

^b^GAD-7: Generalized Anxiety Disorder-7 Scale.

^c^NDDI-E: Neurological Disorders Depression Inventory for Epilepsy.

### Primary Outcome

Both groups improved over time, showing significant within-group improvements in the QOLIE-31 total score compared with baseline (*P*<.001). Effect sizes were medium for the control group (Hedges *g=*0.57, 95% CI 0.29-0.86) and large for the intervention group (Hedges *g=*1.38, 95% CI 1.07-1.68; Table 2). The linear mixed model also indicated that the interactions between the intervention and follow-up time were statistically significant (*P*<.001; Table 2); the observed (between-group) effect size was small (Hedges *g=*0.49, 95% CI 0.22-0.76; Table 2).

**Table 2:** Results of intention-to-treat (ITT) outcome measures.

Measure			Before the intervention, mean (SD)	After the intervention, mean (SD)	Within-group effect sizes, Hedges *g* (95% CI)	*P* value	*F* (Group× time)^a^	*P* value (Group× time)^a^	Between-group effect sizes, Hedges g (95% CI)
**Primary outcome: QOLIE-31^b^ total score**									
	Intervention (N=107)		65.22 (7.21)	75.02 (6.94)	1.38 (1.07-1.68)	<.001	13.10	<.001	0.49 (0.22-0.76)
	Control (N=107)		65.23 (8.09)	69.79 (7.16)	0.57 (0.29-0.86)	<.001	N/A^c^	N/A	>N/A
**Secondary outcome**									
	**Seizure worry**								
		Intervention (N=107)	58.33 (13.72)	77.27 (18.49)	1.17 (0.87-1.47)	<.001	10.18	<.001	0.43, (0.16-0.70)
		Control (N=107)	59.01 (13.82)	68.86 (13.72)	0.74 (0.46-1.03)	<.001	N/A	N/A	N/A
	**Overall quality of life**								
		Intervention (N=107)	72.21 (11.35)	83.80 (16.02)	0.35 (0.07-0.63)	<.001	10.20	.01	0.43 (0.16-0.70)
		Control (N=107)	75.33 (30.29)	81.99 (30.72)	0.12 (0.01 to 0.42)	<.001	N/A	N/A	N/A
	**Emotional well-being**								
		Intervention (N=107)	65.42 (8.69)	80.06 (10.10)	1.54 (1.22-1.85)	<.001	26.51	<.001	0.70 (0.43 to 0.98)
		Control (N=107)	64.21 (9.85)	68.63 (10.63)	0.45 (0.17-0.73)	<.001	N/A	N/A	N/A
	**Energy or fatigue**								
		Intervention (N=107)	60.13 (10.37)	74.14 (10.98)	1.28 (0.98-1.59)	<.001	18.74	<.001	0.58 (0.32-0.86)
		Control (N=107)	60.40 (10.51)	65.15 (10.56)	0.47 (0.19-0.75)	<.001	N/A	N/A	N/A
	**Cognition**								
		Intervention (N=107)	64.94 (11.96)	69.19 (11.74)	0.37 (0.09-0.65)	<.001	.083	0.57	0.04, (–0.23 to 0.31)
		Control (N=107)	64.29 (12.21)	67.97 (11.31)	0.30 (0.03-0.58)	<.001	N/A	N/A	N/A
	**Medication side effects**								
		Intervention (N=107)	53.67 (14.63)	79.57 (14.50)	1.74 (1.42-2.07)	<.001	33.89	<.001	0.79 (0.52-1.07)
		Control (N=107)	50.07 (13.90)	58.68 (15.85)	0.56 (0.28 to 0.84)	<.001	N/A	N/A	N/A
	**Social function**								
		Intervention (N=107)	66.18 (9.02)	72.07 (10.48)	0.59 (0.30 to 0.87)	<.001	1.91	.09	0.19 (–0.08 to 0.46)
		Control (N=107)	65.49 (10.91)	69.40 (10.44)	0.34 (0.06-0.62)	<.001	N/A	N/A	N/A
	**GAD-7^d^**								
		Intervention (N=107)	7.43 (5.95)	6.76 (5.06)	–0.22 (–0.14 to –0.09)	.008	.493	.12	010 (–0.17 to 0.36)
		Control (N=107)	7.17 (5.28)	5.70 (4.55)	–0.29 (–0.57 to –0.01)	<.001	N/A	N/A	N/A
	**NDDI-E^e^**								
		Intervention (N=107)	13.04 (5.63)	10.21 (4.286)	–0.57 (–0.85 to –0.29)	<.001	9.28	.01	0.42 (0.15-0.68)
		Control (N=107)	12.18 (5.10)	10.63 (4.4.40)	–0.32 (–0.60 to –0.04)	<.001	N/A	N/A	N/A

^a^Results of ITT outcome analysis using linear mixed models.

^b^QOLIE-31: Quality of Life in Epilepsy-31.

^c^N/A: not applicable.

^d^GAD-7: Generalized Anxiety Disorder-7 Scale.

^e^NDDI-E: Neurological Disorders Depression Inventory for Epilepsy.

As for the 7 subdomain scores of QOLIE-31, both groups improved over time, showing significant within-group improvements in 7 domains scores compared with baseline (all *P*<.001). The effect sizes for the control group were small (eg, overall quality of life, emotional well-being, energy or fatigue, cognition, and social function) and medium (eg, seizure worry, medication side effects). The effect sizes for the intervention group were small (eg, overall quality of life, cognition), medium (eg, social function), and large (eg, seizure worry, emotional well-being, energy or fatigue, and medication side effects). Refer to Table 2 for further details. For all 7 subdomain scores of QOLIE-31, except cognition (*P*=.57) and social activities (*P*=.09) subdomains, the interactions between the intervention and time remained statistically significant (all *P*<.05). Refer to Table 2 for further details. The effect sizes of 7 subdomains scores of QOLIE-31 between groups after the intervention were small (eg, seizure worry, overall quality of life) and medium (eg, emotional well-being, energy or fatigue medication side effects). Refer to Table 2 for further details. The more detailed linear mixed model results are documented in Multimedia Appendix 2.

### Secondary Outcomes

Both groups improved over time, showing significant within-group reductions in GAD-7 score compared with baseline (*P*<.05). Effect sizes were small for both groups (control group, Hedges *g*=–0.29, 95% CI –0.57 to –0.01, intervention group, Hedges *g*=–0.22, 95% CI –0.14 to –0.09; Table 2). The linear mixed model indicated that the interactions between the intervention and follow-up time were not statistically significant (*P*=.12; Table 2).

Both groups improved over time, showing significant within-group reductions in NDDI-E score compared with baseline (*P*<.001). Effect sizes were small for the control group (Hedges *g*=–0.32, 95% CI –0.60 to –0.04) and medium for the intervention group (Hedges *g*=–0.57, 95% CI –0.85 to –0.29; Table 2). The linear mixed model also indicated that the interactions between the intervention and follow-up time were statistically significant (*P*=.01; Table 2); the observed (between-group) effect size was small (Hedges *g*=0.42, 95% CI 0.15 to 0.68; Table 2).

## Discussion

### Principal Findings and Comparison With Prior Work

This study aims to compare IH follow-up management with traditional outpatient follow-up in terms of quality of life, anxiety, and depression among patients with epilepsy. The results indicated that, except for the cognitive and social function dimensions, the improvements in the total QOLIE-31 scale score (Hedges *g*=0.49) and other dimension scores for patients with epilepsy under IH follow-up management were significantly greater compared to those under hospital outpatient follow-up management. Greater reductions in the depression were observed at 6 months after randomization in the intervention group than in the control group (Hedges *g*=0.42).

The findings of this study indicate that both hospital outpatient follow-up and IH follow-up significantly improved the quality of life and reduced anxiety and depression in people with epilepsy. However, it is important to note that in the assessment of anxiety using the GAD-7, while the effect of time was significant, the difference between groups was not statistically significant. This suggests that the observed improvement in anxiety levels may have been influenced, at least in part, by natural changes over time rather than solely by the intervention itself. Therefore, caution should be exercised in interpreting this result, and future studies should use more rigorous research designs to further verify the specific effects of internet-based follow-up.

As a chronic neurological disorder, epilepsy requires a strong focus on standardized long-term management. Effective management involves multiple components, including health education, seizure monitoring, treatment adjustments, monitoring drug side effects, and addressing comorbidities [22]. Successful management depends on collaboration between patients and health care providers to establish a sustainable long-term follow-up relationship. Epilepsy management can be approached through different follow-up models. Traditional outpatient follow-up is the most common, but emerging models, such as using platforms like WeChat for long-term management, have also gained attention. For example, studies like that of Tong Qianxi [14] have demonstrated improved treatment adherence in pediatric patients with epilepsy through such platforms, highlighting their potential for long-term management. The model used in this study combines outpatient care, either traditional or internet-based, with comprehensive epilepsy health education, addressing not only medical needs but also empowering patients with knowledge for better self-management.

Both outpatient and IH follow-up, when coupled with health education, resulted in improved quality of life for patients with epilepsy. Health education plays a crucial role in epilepsy management. Ridsdale et al [23] conducted health education programs focusing on self-management for patients with epilepsy. Their study demonstrated that, after 12 months, patients showed improved quality of life and alleviated symptoms of anxiety and depression. Health education helps patients understand the causes and progression of epilepsy, treatment options, and daily care strategies [24]. With a better understanding of the disease, patients and their families are better equipped to respond rationally, take appropriate treatment measures, reduce unnecessary panic and anxiety, and enhance their confidence in managing the disease [25].

In this study, the regular follow-up model, combining either outpatient care (hospital-based or IH) with epilepsy health education, led to improvements in quality of life, anxiety, and depression. The management of chronic diseases, including epilepsy, is a key area of ongoing research. Hospital-based models, particularly those involving multidisciplinary epilepsy teams, are best suited for patients with severe epilepsy [26]. Another approach involves community-centered models, which provide an alternative for managing epilepsy in areas with limited medical resources. However, these models may be constrained by economic, regional, and privacy factors [27]. In contrast, IH follow-up, coupled with health education, addresses key challenges such as time constraints and geographic limitations. This model offers an accessible, sustainable option for long-term management, particularly for patients with stable epilepsy (non-DRE) who may benefit from continuous monitoring and support without the need for frequent in-person visits.

The results of this study demonstrated that IH follow-up management, as a medical service model, led to significant improvements in the overall quality of life score, as well as reductions in anxiety among patients with epilepsy, compared to traditional outpatient follow-up management, suggesting that IH follow-up is an effective and feasible approach for chronic disease management in patients with epilepsy. This is similar to previous research on IH models for chronic disease management, such as studies focused on diabetes and hypertension, which have also reported positive outcomes [28,29]. This may be because IH follow-up improves patients' adherence to treatment plans through remote prescriptions, medication reminders, and health education, reducing epilepsy seizures caused by missed doses or unauthorized medication adjustments. Traditional offline follow-up is often limited by time and space, making it difficult for patients to receive timely medical guidance when their symptoms change between visits. IHs enable patients to access professional medical advice more quickly through remote consultations and web-based inquiries, reducing unnecessary medical actions caused by misinformation or anxiety. Prior studies have explored internet-based platforms for epilepsy care; most have focused on specific tools, such as mobile health applications [30]; however, our study provides an in-depth analysis of the unique role of IHs.

While IH follow-up offers several advantages, the traditional outpatient follow-up model still remains prevalent. It faces several significant challenges, such as information asymmetry, high time costs, difficulties securing appointments with specialists, and limited accessibility for patients in remote areas. IHs address these issues by offering a more convenient option for patients who are constrained by time or geographic location [31]. For patients with epilepsy with relatively stable conditions who require long-term medication management, frequent hospital visits for prescriptions are often hindered by time and logistical barriers, leading to higher accommodation and transportation costs. IHs offer a more efficient alternative by enabling real-time symptom reporting, personalized treatment consultations, and immediate prescription refills, which are crucial for managing epilepsy and improving patient adherence to long-term medication regimens. This eliminates the inconvenience and delays caused by travel, long waiting times, and geographic constraints [32]. Consequently, it enhances the overall convenience and efficiency of medical services, reduces travel-related fatigue, and lessens the financial burden on patients. Additionally, the timeliness of IH care alleviates concerns about the inability to consult doctors promptly in case of changes in the patient's condition or adverse drug reactions. However, no significant improvements were observed in the cognitive and social function dimensions for patients managed through IH follow-up compared to those managed through traditional outpatient follow-up. The improvement of cognitive and social function relies on long-term environmental stimulation, psychological support, and real-life social interactions, which IH follow-up cannot fully provide. Additionally, cognitive and social function improvement is often closely related to changes in the patient's condition (eg, epilepsy seizure frequency), and such changes may not show significant improvements within a short-term follow-up period.

For newly diagnosed patients with epilepsy or those experiencing significant changes in their condition, face-to-face consultations are often necessary. For these non-DREs, whose conditions are relatively stable, follow-up through IHs is more suitable. Epilepsy requires comprehensive evaluation, including physical examination, detailed medical history inquiry, assessment of the patient’s overall health, and relevant tests to formulate a tailored diagnosis and treatment plan, which cannot be fully replaced by IHs or other telemedicine methods.

### Limitations

The follow-up period in this study was 6 months. While this duration was sufficient to assess the short-term impact of internet-based follow-up on the quality of life, anxiety, and depression in patients with epilepsy, it may not fully capture long-term trends or impacts on cognitive and social functions. Future studies could consider extending the follow-up period to at least 1 year or longer to explore the long-term effects of internet-based follow-up on cognitive and social function in patients with epilepsy. Additionally, longer follow-up could help reveal the sustained impact of internet-based follow-up on patients' quality of life and whether there are fluctuations or improvements over time.

We have explicitly acknowledged the limitations of using self-reported measures, such as the GAD-7 and NDDI-E, for assessing anxiety and depression in patients with epilepsy. While these tools are validated and widely used, they rely on patient self-reporting, which can be influenced by subjective factors such as patients' emotional state, social desirability bias, or misunderstanding of the questions.

We also acknowledge that differences in nurse experience may have contributed to the observed differences, with 1 nurse having 10 years of experience in epilepsy specialty care and the other having 8 years. These factors should be further explored and controlled for in future research. Additionally, we have noted that the IH follow-up group may have received extra web-based educational materials, which could introduce potential confounding effects, particularly in terms of educational support and access to information. To ensure a more equitable comparison between the 2 groups, future research should consider balancing non-digital support, such as providing similar educational materials and support resources to the traditional outpatient group.

This study was conducted at a single hospital, which introduces selection bias and limits its generalizability. Furthermore, cultural factors may influence patients' willingness to adopt IH services or their expectations of health care. Therefore, future research should explore the application of IH models in diverse settings to assess their adaptability and effectiveness across different health care systems and cultural contexts.

While IHs offer many advantages, they also face challenges in implementation, particularly among resource-limited populations or those with low digital literacy. For example, patients may lack the skills to use the internet or face device limitations, which can impact their engagement and outcomes. Additionally, data security and privacy issues are critical concerns that IHs need to address. Therefore, future research should consider ways to improve digital literacy, ensure the availability of devices, and strengthen data protection.

While the study received ethical approval and participant consent, we acknowledge potential biases, particularly related to participants' access to technology. Disparities in internet service availability may affect patient participation. Future research should ensure equal access to internet resources and control for differences in service availability.

### Conclusions

Regular follow-up management through either IHs or hospital outpatient clinics can improve the quality of life for patients with epilepsy and alleviate their anxiety and depression. For patients with epilepsy managed through IH follow-up, their quality of life and depression are more likely to improve. Therefore, IH follow-up management is recommended for wider use in the follow-up care of patients with epilepsy. In clinical practice, it is essential not only to focus on controlling seizures and reducing related harm but also to thoroughly understand the patient's emotional state and provide proactive health education, along with necessary medication treatment.

## Appendix

Multimedia Appendix 1 CONSORT-eHEALTH checklist (V 1.6.1).

Multimedia Appendix 2 Additional analyses and statistics.

## Abbreviations

CONSORT: Consolidated Standards of Reporting Trials
DRE: drug-resistant epilepsy
GAD-7: Generalized Anxiety Disorder-7 Scale
IH: internet hospital
MOSES: Modular Service Package Epilepsy
NDDI-E: Neurological Disorders Depression Inventory for Epilepsy
QOLIE-31: Quality of Life in Epilepsy-31

## Data Availability

The datasets generated and analyzed during the present study are not publicly available due to the lack of participant consent for public sharing. However, the data can be obtained from the corresponding author upon reasonable request.
