# Supplementary material for: The Impact of Internet Hospital Follow-Up on the Quality of Life of Patients With Epilepsy: Randomized Controlled Trial
Source: J Med Internet Res. 2025 May 26;27:e70665. doi: 10.2196/70665 (PMC12149770; doi:10.2196/70665)
Supplement: Multimedia Appendix 2 [file jmir_v27i1e70665_app2.docx]

| **Table S1.** Results of intention-to-treat (ITT) outcome analysis using linear mixed models | | | | | |
| --- | --- | --- | --- | --- | --- |
| Outcome | β estimate (SE; 95% CI) | *P* value | Outcome | *β* estimate (SE; 95% CI) | *P* value |
| **QOLIE-31**^a^ **Total score** | | | **Cognition** | |  |
| Intercept | 65.20 (N/A) ^b^ | N/A | Intercept | 64.06 (N/A) | N/A |
| Group | 0.20 (1.04, -1.85 to 2.26) | .84 | Group | 1.37 (1.65, -1.88 to 4.62) | .41 |
| Time | 4.463(0.67, 3.11 to 5.82) | <.001 | Time | 3.63(0.87, 1.91 to 5.35) | <.001 |
| Group×time | 5.341(0.97, 3.42 to 7.26) | <.001 | Group×time | 0.67(1.24, -1.76 to 3.11) | 0.57 |
| **Seizure worry** | |  | **Medication side effects** | |  |
| Intercept | 59.23 (N/A) | N/A | Intercept | 50.64 (N/A) | N/A |
| Group | -0.59(2.11, -4.74 to 3.56) | .78 | Group | 3.51(2.10, -0.63 to 7.64) | .10 |
| Time | 9.95(1.32, 7.34 to 12.55) | <.001 | Time | 8.49(1.59, 5.36 to 11.61) | <.001 |
| Group×time | 9.51(1.87, 5.82 to 13.20) | <.001 | Group×time | 17.30(2.25, 12.87 to21.73) | <.001 |
| **Overall quality of life** | |  | **Social Function** | |  |
| Intercept | 72.73 (N/A) | N/A | Intercept | 65.61 (N/A) | N/A |
| Group | -0.81 (1.67, -4.10 to 2.49) | .63 | Group | 0.70(1.42, -2.10 to 3.49) | .63 |
| Time | 6.40 (1.42, 3.61 to 9.19) | <.001 | Time | 3.54(0.90, 1.77 to 5.31) | <.001 |
| Group×time | 5.06(2.01, 1.11 to 9.02) | .01 | Group×time | 2.190(1.27, -0.32 to 4.50) | .09 |
| **Emotional well- being** | |  | **GAD-7**^c^ | |  |
| Intercept | 63.80 (N/A) | N/A | Intercept | 7.09 (N/A) | N/A |
| Group | 1.51(1.39, -1.24 to 4.25) | .28 | Group | 0.45(0.74, -1.01 to 1.91) | .54 |
| Time | 4.62(0.85, 2.94 to 6.30) | <.001 | Time | -1.46(0.33, -2.11 to -0.79) | <.001 |
| Group×time | 10.14(1.21, 7.76 to 12.53) | <.001 | Group×time | 0.74 (0.47, -0.20 to 1.67) | .12 |
| **Energy/fatigue** | |  | **NDDIE**^d^ | |  |
| Intercept | 60.07 (N/A) | N/A | Intercept | 12.19 (N/A) | N/A |
| Group | 0.18(1.50, -2.77 to 3.13) | .902 | Group | 1.05(0.69, -0.31 to 2.42) | .13 |
| Time | 4.91(0.95, 3.03 to 6.79) | <.001 | Time | -1.54 (0.37, -2.26 to -0.81) | <.001 |
| Group×time | 9.17(1.35, 6.51 to 11.84) | <.001 | Group×time | -1.37(0.52, -2.39 to -0.34) | .01 |
| ^a^ QOLIE-31: Quality of Life in Epilepsy -31 | | | | | |
| ^b^ N/A: not applicable. | | | | | |
| ^c^ GAD-7: Generalized Anxiety Disorder-7 | | | | | |
| ^d^ Neurological Disorders Depression Inventory for Epilepsy (NDDI-E) | | | | | |
